# The longitudinal relationship between emotion awareness and internalising symptoms during late childhood

**DOI:** 10.1007/s00787-012-0267-8

**Published:** 2012-03-31

**Authors:** Carolien Rieffe, Mark De Rooij

**Affiliations:** Department of Psychology, Leiden University, Leiden, The Netherlands

**Keywords:** Depression, Anxiety, Fear, Worry, Rumination, Emotion regulation, Alexithymia, Adolescents

## Abstract

Emotion awareness, the ability to reflect upon the own emotions, is assumed to contribute to better mental health. However, empirical support for this relationship has only been cross-sectional. In this study we examined the extent to which individual differences in changes in emotion awareness over time can explain individual differences in changes in symptoms of internalising problems (depression, fear, worrying and ruminative thoughts). Children and young teenagers (368 boys and 295 girls) were asked four times to fill out self-report questionnaires, with a 6-month time interval between each time. The mean age was 10 years during the first data collection. Longitudinal multilevel analyses showed that the variance in emotion awareness trends was highly predictive for the variance in trends for internalizing problems over time. The ability to differentiate discrete emotions was a strong predictor and negatively contributed to all internalising symptoms. In addition, a diminished tendency to address and value emotions contributed to more depressive symptoms; whereas hiding the own emotions contributed to more worrying and ruminative thoughts. The outcomes show that individual differences in emotion awareness over time make a strong, and, above all, negative contribution to the prediction of the individual differences in various internalizing symptoms. The fact that several aspects of emotional (dys)functioning are uniquely related to different kinds of internalizing problems gives valuable and useful information not only theoretically but also clinically about the distinctive nature of these problems.

## Introduction

Basic emotions entail functional response programmes that serve to quickly adapt to changes in the environment [13]. Yet, in today’s Western societies, we seldom encounter direct life-threatening situations that require such a swift response pattern, and—in contrast—our daily social interactions benefit more from a certain level of emotional control. In fact, our social contacts on a day to day basis are not very lenient toward a blunt expression of these initial impulses. Thus, in order to manage these primary responses, children learn from very early on how to modify their emotions and express them in socially acceptable ways. In other words, children are taught how to find a balance between their own desires and needs on the one hand, and goals and demands from society on the other, without jeopardising their social relations. This process of emotion socialisation leads in normal development to an emotion control that becomes more or less automated, like using the gear while driving a car [23].

However, a certain degree of attention to and insight in the own emotional responses and functioning is still necessary, because this insight is regarded to be a prerequisite with respect to effective emotion regulation [11]. This capacity is what we refer to as emotion awareness. Impairments in emotion awareness are associated with higher levels of internalising problems such as symptoms of depression or anxiety (e.g. [19]). The aim of this study is to examine the frequently assumed causality of these associations between emotion awareness and different internalising problems. Internalising problems, such as depression and anxiety, show high co-morbidity [2, 28]. Impairments in emotion awareness may be at the root of both disorders. Understanding the extent to which various aspects of emotion awareness uniquely contribute to the prediction of children’s self-reported internalising problems will improve our knowledge of the convergences and divergences in the etiology of these internalising problems.

### Emotion awareness

By definition, an emotion is related to an external event that requires a quick and adaptive reaction. As Scherer (p. 152) [23] puts it, an emotion is “a hypothetical construct denoting a process of an organism’s reaction to significant events”. In contrast with the widespread, so called James–Lange notion that emotions occur through detection of the bodily arousal during the emotion episode, we support the notion that an emotion experience can only arise in its situational context [7]. In line with the appraisal theory, we state that emotion awareness requires an external focus, i.e. a focus on oneself in relation to the emotion evoking situation [12]. Consequently, emotion awareness can be defined as an attentional process that serves to monitor and differentiate the own discrete emotions, which is strongly related to the ability to locate their antecedents, and in which, in fact, little attention is paid to the physical arousal that is part of the emotion experience. In addition, emotion awareness includes attitudinal aspects, such as how emotion experiences are valued in one self and others and how the own emotions should be expressed or communicated [19].

Especially, the ability to differentiate discrete emotions, also a key factor in the related but more narrowly defined concept of alexithymia, is known to be strongly related to better emotion regulation [3] and fewer symptoms of mental problems, such as depression and anxiety [26]. Several studies confirm that also in elementary school age children, a higher level of alexithymia and other problems in the domain of emotion awareness are related to internalising problems, such as depression, fear or anxiety, somatic complaints, worry and rumination [16–19, 21].

These previous studies have consistently shown that there were three major attentional aspects of emotion awareness (i.e. the inability to differentiate between emotions and talk about them, and a stronger awareness of bodily symptoms during an emotion experience) that were unique contributors to the prediction of various internalising symptoms. Impairments in these aspects of emotion awareness denote an internally self-oriented focus instead of an externally oriented focus that is aimed at understanding and adaptively solving the emotion-evoking situation. Probably, a self-oriented focus limits one’s ability to thoroughly analyse the emotion evoking event and identify the different aspect in the situation that could call for different action tendencies and related emotion experiences. Consequently, the (more global and less well defined) emotion experience will linger on and might eventually be detached from the actual evoking situation [23]. Also an unwillingness to address the own emotions—an attitudinal aspect of emotion awareness—was related to more frequent symptoms of depression in these studies [19], whereas the tendency to hide the emotions from others was related to more social anxiety and worrying thoughts in young adolescents [19, 21].

To date, however, studies that have confirmed associations between the various aspects of emotion awareness and internalising symptoms have been based on cross-sectional data collections. Although there is a credible theoretical framework to underline the frequently assumed causality of impaired emotion awareness on the development of internalising symptoms, this has not yet been studied longitudinally. Confirmation of this theoretical framework, not only cross-sectional but also longitudinal, is in line with a current trend in the literature stressing that internalising symptoms frequently co-occur and it is important for both academics and practitioners to better understand their convergences and divergences [2, 28].

### Current study

The aim of this study was to examine the extent to which individual differences in changes in emotion awareness over time can explain individual differences in changes in symptoms of internalising problems during late childhood, i.e. in a population of elementary school-aged children. The age period was chosen, because at this age, cognitive control mechanisms become increasingly important, and children become progressively more able to reflect upon their own internal states and emotions [8]. The internalising symptoms included in this study reflect the most common symptoms in late childhood and young adolescents: depression, fear, and worrying and ruminative thoughts [1, 22].

Based on previous findings [16, 17, 21], it was expected that an inability to differentiate between emotions and locate their antecedents (Differentiation), talk about them (Verbal Sharing) and a stronger focus on the bodily symptoms that are part of the emotion experience (Bodily Symptoms) would significantly contribute to the prediction of all three internalising symptoms in this study (Depression, Fear, Worry/Rumination). In addition, it was expected that an unwillingness to address the own emotions (Own Emotions) would contribute to the prediction of depression, whereas the tendency to hide one’s emotions (Not Hiding) was expected to contribute to the prediction of fear and repetitive, unconstructive (anticipatory) thoughts about a negative event (Worry/Rumination). Attention to other people’s emotions (Others’ Emotions) previously showed significance in young adolescents only, but not in elementary school children [16]. Since the sample in this study consisted of elementary school children, it was expected that a stronger willingness to pay attention to and understand other people’s emotions would be unrelated to internalising symptoms in this study at this age.

Gender was taken into account, but no hypotheses could be formulated based on the existing literature, except for the expectation that girls would report more frequent internalising symptoms, which is evident in all studies that are based on large community samples [6].

## Method

### Participants and procedure

A total of 717 children participated in this study, but the data of 663 children were complete and used for the current data analyses (54 children missed one or more sessions for several reasons, e.g. absence during the day of testing, or the family had moved). Children were drawn from seven different primary schools in the larger area of Den Bosch, The Netherlands and came predominantly from middle class backgrounds. The group for the current study consisted of 368 boys (mean age 10 years, 3 months; SD = 8 months) and 295 girls (mean age 10 years, 3 months; SD = 9 months) during the first data collection. Parental consent was obtained prior to the data collection for all participants.

Children were handed out questionnaires in class and asked to fill these out after the experimenter had given instructions. The testing took approximately 1 h. The full study included more questionnaires than the ones that were used for the analyses in this paper. Children were asked to fill out these questionnaires four times, with a 6-month-time interval between each time. The Emotion Awareness Questionnaire (EAQ30), Children’s Depression Inventory (CDI) and Worry/Rumination Questionnaires were completed four times. However, the Fear Schedule was administered three times. The Fear Schedule was not included at Time 3.

## Materials

The EAQ30 [19] aims to identify how children and adolescents feel and think about their feelings. The Emotion Awareness Questionnaire (30 items of which 20 items are negatively formulated and thus reversed-scored) was designed with a 6-factor structure describing six aspects of emotional functioning, thus containing six scales: (1) Differentiating Emotions (e.g. ‘When I am upset, I do not know if I am sad, scared or angry’, reversed-scored); (2) Verbal Sharing of Emotions (e.g. ‘I can easily explain to a friend how I feel inside’); (3) Not Hiding Emotions (e.g. ‘When I am upset, I try not to show it’, reversed-scored); (4) Bodily Awareness of Emotions (e.g. ‘When I feel upset, I can also feel it in my body’, reversed-scored); (5) Attending to Others’ Emotions (e.g. ‘If a friend is upset, I try to understand why’); (6) Analyses of Emotions (e.g. ‘My feelings help me to understand what has happened’). Respondents are asked to rate the degree to which each item is true about them on a three-point response scale (1 = not true, 2 = sometimes true, 3 = often true). In all the scales, a higher score represented a higher presence of this ability, with the exception of Bodily Awareness, in which a higher score implies less attention to bodily symptoms. The scales have good internal consistencies [19, 21], which was confirmed in this study (Table 1).

**Table 1:** Internal consistencies of the questionnaires for Depression, Fear, Worry/Rumination, and the six scales of the Emotion Awareness Questionnaire

	No. of items	Cronbach’s alpha	Inter-item correlation
Depression	26	0.81	0.15
Fear	80	n.a.	0.28
Worry/Rumination	10	0.85	0.36
Emotion Awareness			
Differentiating	7	0.75	0.30
Verbal Sharing	3	0.68	0.42
Not Hiding Emotions	5	0.74	0.36
Bodily Awareness	5	0.60	0.22
Others’ Emotions	5	0.70	0.31
Analyses Emotions	5	0.78	0.42

The CDI ([10]; Dutch translation: [27]) is a widely used self-report questionnaire that assesses cognitive, affective and behavioural signs of depression in children and adolescents from 6 to 17 years old. The CDI contains 26 items, each of which consists of three statements. For example ‘I have fun doing most things’, ‘I have fun doing some things’ and ‘Nothing much is fun for me’. Participants are asked to select the statement that best describes their feelings in the past 2 weeks. The mean sum score is used in this study. The Depression Inventory has a good internal consistency [14, 19], which was confirmed in this study (Table 1).

The Revised Fear Survey Schedule for Children [15] is a self-report questionnaire, containing 80 items on which children can report the extent to which they fear specific stimuli or situations on a three-point scale (0 = not at all to 2 = very much). The questionnaire has good psychometric properties [15].

The Worry/Rumination Questionnaire for Children [16, 19] reflects the tendency to dwell on a problem instead of dealing with it in terms of solving or coping adaptively with the emotional impact of the situation. The questionnaire comprises 10 items with good internal consistency [16, 17, 21], which was confirmed in this study (Table 1). Respondents are asked to rate the degree to which each item is true about them on a three-point scale (1 = not true, 2 = sometimes true, 3 = often true). The scoring is reversed for one item.

### Statistical analyses

The multilevel model of change [24] was used for the analysis of change in Depression (Worry/Rumination and Fear respectively) with time measured in years and centred around 8.5 years old (the age of the youngest child) as an explanatory variable. First, an unconditional growth model was applied. Second, variation in the intercepts and slopes (trends) was linked to the emotion awareness scales. The Emotion Awareness scales measured at Time 1 were used to explain variation in the intercepts. To establish the association between changes in emotion awareness and changes in Depression (Worry/Rumination and Fear, respectively), we generalized a procedure proposed by Beutler and Hamblin [5], and Steketee and Chambless [25]. In this procedure for change in pre–post test designs, residual gain scores are correlated to establish the association. Residual gain scores are defined as the personal gain scores minus the mean gain scores. We generalized this procedure by fitting an unconditional growth model for each of the Emotion Awareness scales and extracting the slopes for each child. That is, for every Emotional Awareness scale we fitted the following multilevel model, whereby each individual has a unique intercept (*a*_*i*_) and slope (*b*_*i*_)

1 $$ \begin{aligned} & Y_{i,t} = a_{i} + b_{i} \left( {{\text{age}} - 8.5} \right) + e_{i,t} \\ & a_{i} = a + u_{i} \\ & b_{i} = b + v_{i} \\ \end{aligned} $$

where *a*_*i*_ is divided in an overall intercept and a individual deviation *u*_*i*_, this also applies to *b*_*i*_ with the individual deviation *v*_*i*_, and *Y*_*i,t*_ are the measurements of participant *i* at time point *t*. The estimates *v*_*i*_ are generalizations of the residual gain scores and were used as explanatory variables for the slopes of Depression (Worry/Rumination and Fear, respectively). All analyses were performed using MLwin 2.02.

## Results

### Unconditional growth models for the six Emotion Awareness scales

An unconditional growth model was applied to each of the six Emotion Awareness scales to obtain an individual trend. Table 2 shows estimates of the minimum and maximum values of the trends [*v*_*i*_ from formula (1)] as compared to the mean trend. Individual differences in trends were found for Differentiating, Not Hiding, Others’ Emotions and Analysis of Emotions. Although Verbal Sharing and Bodily Awareness showed differences over time, they failed to show individual differences in these trends (all *v*_*i*_ are the same across subjects). Consequently, these two scales could not be used as explanatory variables for changes in Depression, Fear, and Worry/Rumination, since there was no variability.

**Table 2:** Minimum and maximum of estimates of individual trends, *v*_*i*_, from formula (1), in the Emotion Awareness scales

	Differentiating	Verbal Sharing	Not Hiding	Bodily Awareness	Others Emotions	Analyses Emotions
Minimum	−0.13	0	−0.15	0	−0.19	−0.18
Maximum	0.13	0	0.15	0	0.17	0.19

### Growth model for Depression

For Depression, the unconditional growth model with linear trends fits best. In this model the general trend is negative, indicating an overall decline in depression. Furthermore, there is a negative intercept slope correlation, indicating that the children who start high on depression have a stronger negative slope than the children who start low on depression. The variance of the intercepts equals 0.913, while the variance of the slopes equals 0.070. The inclusion of explanatory variables to explain the variability of the intercepts and trends gives a significant improvement of the model (χ^2^ = 177.16, *df* = 11, *p* < 0.05). At age 8.5 (the age of the youngest child at Time 1), Gender and the Emotion Awareness scales explain 32 % of the variance in Depression. In addition, trends in Emotion Awareness explain 49 % of the variation in the slopes. In other words, 49 % of the variation in individual differences over time in Depression can be explained by variation in the Emotion Awareness scales.

Table 3 shows the multi-level regression parameters, i.e. the intercept and slope for each internalizing symptom. Note that the effect of Differentiating on the intercept is established by taking the measurement of Differentiating at Time 1, while the effect of Differentiating on the trend is the effect of the slope of Differentiating on the trend of Depression. To interpret the effects of the Emotion Awareness scales on the change in Depression, consider the following formula:

$$ {\text{Depression}} = \left( {0. 3 3 5+ b_{ 1} \times {\text{ EA}}_{ 1} } \right) + \left( { - 0. 1 3 1+ b_{ 2} \times {\text{ EA}}_{\text{t}} } \right) \times {\text{time}}, $$

where, EA_1_ is an abbreviation of the score on an Emotion Awareness scale at the first time point and EA_t_ is the slope of an Emotion Awareness scale [*v*_*i*_ from formula (1)]. The regression weights *b*_1_ and *b*_2_ reflect the effects of these two variables on the depression score, conditional on all the other variables. More specifically, the regression effect of Differentiating on the intercept (*b*_1_) equals −0.950 (see Table 3) which means that with every unit increase in Differentiating, the Depression score is lowered by −0.950. Furthermore, the effect of the slope of Differentiating on the trend (*b*_2_) equals −2.300. The results in Table 2 show that the minimum slope for Differentiating equals −0.13 and the maximum slope equals 0.13. Consequently, for someone with the minimum trend for Differentiating, the development of Depression over time equals

$$ \left( { - 0. 1 3 1- 2. 300 \times \, - 0. 1 3} \right) \times {\text{time}} = 0. 1 6 8\times {\text{time}}. $$

**Table 3:** Unstandardized regression coefficients (standard errors) for the Emotion Awareness scales on Depression, Fear, and Worry/Rumination at Time 1 (under intercept); and for the slopes of the Emotional Awareness scales on the trends of Depression, Fear, And Worry/Rumination (under trend)

	Depression		Fear		Worry/Rumination	
	Intercept	Trend	Intercept	Trend	Intercept	Trend
Gender	−0.064 (0.119)	−0.030 (0.042)	−0.766 (0.121)**	0.024 (0.039)	−0.259 (0.116)*	−0.004 (0.042)
Differentiating	−0.950 (0.098)**	−2.300 (0.363)**	−0.694 (0.097)**	−1.854 (0.327)**	−1.030 (0.089)**	−2.654 (0.342)**
Verbal Sharing	−0.138 (0.058)*	–	−0.153 (0.057)**	–	−0.230 (0.052)**	–
Not Hiding	0.019 (0.076)	−0.491 (0.267)	−0.111 (0.075)	−0.175 (0.241)	−0.153 (0.069)*	−0.506 (0.252)*
Bodily Awareness	−0.184 (0.076)*	–	−0.225 (0.075)**	–	−0.283 (0.068)**	–
Others Emotions	−0.089 (0.084)	−0.565 (0.212)**	0.026 (0.083)	−0.044 (0.190)	0.082 (0.076)	0.185 (0.197)
Analyses Emotions	−0.393 (0.081)*	−0.716 (0.222)**	0.095 (0.080)	0.206 (0.200)	0.045 (0.073)	−0.167 (0.208)

* *p* < 0.05; ** *p* < 0.01

Yet, for someone with the maximum trend for Differentiating, the development of Depression over time equals

$$ \left( { - 0. 1 3 1- 2. 300 \times 0. 1 3} \right) \times {\text{time}} = - 0. 4 30 \times {\text{time}}. $$

In conclusion, for the first subject (with minimum trend for Differentiating), Depression increased over time; while for the second subject (with maximum trend for Differentiating), Depression decreased over time.

### Growth model for Fear

Also for Fear, the unconditional growth model with linear trends fits best. In this model, the general trend is negative, indicating an overall decline in fear. Furthermore, there is a negative intercept slope correlation. The variance of the intercepts equals 1.713, while the variance of the slopes equals 0.094. The inclusion of explanatory variables to explain the variability of the intercepts and trends gives a significant improvement of the model (χ^2^ = 286.60, *df* = 11, *p* < 0.05). At age 8.5, Gender and the Emotion Awareness scales explain 37 % of the variance in Fear. In addition, the trends in Emotion Awareness explain 25 % of the variation in slopes for Fear. The regression parameters are shown in Table 3. Like for Depression, the formula for Fear is

$$ {\text{Fear}} = \left( {0. 6 5 8+ b_{ 1} \times {\text{ EA}}_{ 1} } \right) + \left( { - 0. 10 2+ b_{ 2} \times {\text{ EA}}_{\text{t}} } \right) \times {\text{time}}. $$

### Growth model for Worry/Rumination

For Worry/Rumination, again the unconditional growth model with linear trends fits best. In this model, the general trend is negative, indicating an overall decline in Worry/Rumination. Furthermore, there is a negative intercept slope correlation. The variance of the intercepts equals 1.196, while the variance of the slopes equals 0.103. The inclusion of explanatory variables to explain the variability of the intercepts and trends gives a significant improvement of the model (χ^2^ = 338.63, *df* = 11, *p* < 0.05). At age 8.5, Gender and the Emotion Awareness scales explain 53 % of the variance in Worry/Rumination. In addition, the trends in Emotion Awareness explain 54 % of the variation in slopes for Worry/Rumination. The regression parameters are shown in Table 3. The formula for Worry/Rumination given the Emotional Awareness variables is

$$ {\text{Worry}}/{\text{Rumination}} = \left( {0. 30 4+ b_{ 1} \times {\text{ EA}}_{ 1} } \right) + \left( { - 0.0 60 + b_{ 2} \times {\text{ EA}}_{\text{t}} } \right) \times {\text{time}}. $$

## Discussion

It is a commonly accepted viewpoint that the strong associations that are repeatedly found between measures indicating insight into the own emotional functioning, such as alexithymia or emotion awareness, with internalizing symptoms, are causal. Again in this study, at a cross-sectional level, we see that the six constructs reflected in the Emotion Awareness Questionnaire have a high predictive value regarding the variance of measures for depression, fear and worrying and ruminative thoughts in children around 9 years old (32, 37 and 54 %, respectively). As in previous findings, an inability to differentiate between various emotions, locate their antecedents, communicate them with others, and pay too much attention to the bodily symptoms of the emotion experience contribute to the prediction of different internalizing symptoms. As stated previously, these outcomes support the view that such an internally and self-oriented focus is on the expense of an externally oriented focus that is aimed at dealing adaptively with the emotion-evoking situation. In addition, in previous studies, a lack of eagerness to understand the own feelings uniquely seemed to contribute to symptoms of depression as in this one; which is also true for the tendency to hide one’s feelings from others with respect to the prediction of worrying and ruminative thoughts [16, 19, 21].

In this study, however, we went beyond the cross-sectional analyses of the data and also examined the extent to which individual differences in the development of internalizing symptoms over four measurements with a 6-month-time interval could be explained by individual differences in the changes of emotion awareness scales over time. Although there was an overall decline for the measures of depression, fear and worrisome thoughts, this was only the case for some children, because other children showed an increase, instead. The outcomes of the longitudinal multilevel analyses showed that the variance in emotion awareness trends was, indeed, highly predictive for the variance in trends for symptoms of depression, fear, and worry or ruminative thoughts over time (49, 25 and 53 %, respectively). The patterns that we found were similar for boys and girls. In this study, we examined the natural development of emotion awareness and internalizing symptoms, where we found trends over time for several variables. However, future studies could also examine intra-individual differences under various situations, as a test for the external validity of the model proposed in this study.

Of the six emotion awareness aspects that were measured with the Emotion Awareness Questionnaire, the scale Differentiating Emotions uniquely contributed to the prediction of all internalizing symptoms. Others’ Emotions and Analyses Emotions contributed to the prediction of depressive symptoms, whereas Not Hiding contributed to the prediction of Worry/Rumination. Unexpectedly, two scales that contributed uniquely to the prediction of all internalizing symptoms at Time 1, Verbal Sharing and Bodily Awareness, showed no individual differences over time, meaning that the changes that occurred over time were the same for all children in the sample. Possibly, this effect occurred because 2 years were too short to statistically notice a change. An alternative explanation could be that these two aspects refer to stable traits, but future studies should examine this issue further. Unfortunately, this lack of individual differences made it impossible to predict the differences in the dependent variables for these two scales, thus the scales were not included in the longitudinal analyses.

These outcomes show that the different aspects of emotion awareness that are represented in the Emotion Awareness questionnaire have predictive value for different internalizing problems. Yet, the ability to differentiate between emotions and understand their causes, which is also a core feature of alexithymia, appears to be a key factor with high predictive value for all internalizing problems that were measured in this study. Children who report an improved ability to differentiate the own emotions and understand their causes also report fewer symptoms of depression, fear and worrying or ruminative thoughts over time. This ability to differentiate denotes that the child is first of all focused on the outside world, or more precisely, tries to analyze the emotion-evoking event more thoroughly. Second, it also implies that the child becomes increasingly aware or able to understand that emotions are not simply “happening”, i.e. one is not the victim of the own emotions, but instead, they are related to a situation which needs to be dealt with.

Regarding depression, besides an improved ability to differentiate between emotions, children who also pay increased attention to the own emotions and those of other report fewer symptoms of depression over time. In other words, children who increasingly understand that emotions give important information that might help to adequately react in social situations, and thus this information should not be ignored, report fewer feelings of a chronically low mood state, in which depression basically consists of. This underlines the frequently made assumption that negative emotions that are not adequately dealt with, result in a so-called ‘emotion residue’ (i.e. affective feelings that no longer have a direct connection to the outside world, the emotion evoking event, that caused the initial emotional state), can contribute to depressive feelings in the long term. Alternatively, or perhaps in addition, a lack of adaptive emotion regulation strategies might mediate this process [3].

Concerning worrying and ruminative thoughts, the outcomes show that besides an improved ability to differentiate between emotions, children who increasingly try to hide feelings from others also report more recurrent negative thoughts over time. Trying to hide one’s feelings—possibly because one is not very able to understand them—implies a less open attitude in social interactions, which might lead to more negative ruminative thoughts, because this attitude prevents others from reacting empathically or offering social support. This relationship might be moderated by the degree of self-esteem, which has also been shown to be related to more worry in adolescents [9].

In summary, the outcomes of this study show that individual differences in emotion awareness over time make a strong and, above all, negative contribution to the prediction of the individual differences in various internalizing symptoms. Furthermore, it opens the possibility to examine which specific elements of emotional (dys)functioning are related to different kinds of psychological problems, which could give clinicians and other professionals a better understanding of the nature of these problems. Although not the focus of this study, these outcomes might also be inspiring to further explore other mental health problems in this direction. For example, the internal self-oriented focus that prevents the individual from dealing adaptively with the external event causing the emotional experience, might be a strategy for whom the emotion experience is too intense and causing over-arousal, as might be the case for individuals with PTSD [4] or children with ASD [20].

Note, however, that there are two limitations to this study that should be kept in mind while interpreting these results. First, this study was based on self-report questionnaires. Future designs could include different measures other than structured self-reports, such as clinical interviews using open-ended questions, to further validate the outcomes of the current study. Second, the time span in this study (four time points with 6-month intervals) covers the younger teenager years. Most internalizing symptoms are known to increase rapidly when children start their boisterous adolescent years; the outcomes of this study raise the question of whether a higher level of emotion awareness will still be protective then, or perhaps other factors become more dominant, such as their hormonal, neurological and other biological changes. Future studies could tackle this question, but also the other assumptions made in the above discussion to explain the outcomes, and include other variables such as emotion regulation strategies or self-esteem.

## References

[1] Aggleton P, Hurry J, Warwick I (2000). Young people and mental health.

[2] Anderson ER, Hope DA (2008). A review of the tripartite model for understanding the link between anxiety and depression in youth. Clin Psychol Rev.

[3] Barrett FL, Gross J, Christensen TC, Benvenuto M (2001). Knowing what you are feeling and knowing what to do about it: mapping the relation between emotion differentiation and emotion regulation. Cogn Emot.

[4] Beevers CG, Lee HJ, Wells TT, Ellis AJ, Telch MJ (2011). Association of predeployment gaze bias for emotion stimuli with later symptoms of PTSD and depression in soldiers deployed in Iraq. Am J Psychiatry.

[5] Beutler LE, Hamblin DL (1986). Individualized outcome measures of internal change: methodological considerations. J Consult Clin Psychol.

[6] Costello EJ, Mustillo S, Erkanli A, Keeler G, Angold A (2003). Prevalence and development of psychiatric disorders in childhood and adolescence. Arch Gen Psychiatry.

[7] Frijda N (1986) The emotions. Cambridge University Press, Cambridge

[8] Harris PL (1989). Children and emotions: the development of psychological understanding.

[9] Kaufman KL, Brown RT, Graves K, Henderson P, Revolinski M (1993). What, me worry: a survey of adolescents concerns. Clin Pediatr.

[10] Kovacs M (1985). The Children’s Depression Inventory (CDI). Psychopharmacol Bull.

[11] Lambie JA, Marcel AJ (2002). Consciousness and the varieties of emotion experience: a theoretical framework. Psychol Rev.

[12] Lazarus RS (1991). Progress on a cognitive–motivational–relational theory of emotion. Am Psychol.

[13] Levenson RW (1999). The interpersonal functions of emotion. Cogn Emot.

[14] Meerum Terwogt M, Rieffe C, Miers AC, Jellesma FC, Tolland A (2006). Emotions and self-esteem as indicators of somatic complaints in children. Infant Child Dev.

[15] Ollendick TH (1983). Reliability and validity of the revised fear survey schedule for children (FSSC-R). Behav Res Therapy.

[16] Rieffe C, Meerum Terwogt M, Petrides KV, Cowan R, Miers AC, Tolland A (2007). Psychometric properties of the Emotion Awareness Questionnaire for children. Personality Individ Differ.

[17] Rieffe C, Oosterveld P, Meerum Terwogt M (2006). An alexithymia questionnaire for children: factorial and concurrent validation results. Personality Individ Differ.

[18] Rieffe C, Oosterveld P, Meerum Terwogt M, Novin S, Nasiri H, Latifian M (2010). Relationship between alexithymia, mood and internalizing symptoms in children and young adolescents: evidence from an Iranian sample. Personality Individ Differ.

[19] Rieffe C, Oosterveld P, Miers AC, Meerum Terwogt M, Ly V (2008). Emotion awareness and internalising symptoms in children and adolescents: the Emotion Awareness Questionnaire revised. Personality Individ Differ.

[20] Rieffe C, Camodeca M, Pouw LBC, Lange AMC, Stockmann L (2012) Don’t anger me! Bullying, victimisation, and emotion dysregulation in young adolescents with ASD. Eur J Dev Psychol (to appear)

[21] Rieffe C, Villanueva L, Adrián JE, Górriz AB (2009). Somatic complaints, mood states, and emotion awareness in adolescents. Psicothema.

[22] Rutter M, Smith D (1995). Psychosocial disorders in young people: time trends and their causes.

[23] Scherer KR, Hewstone M, Stroebe W (2000). Emotion. Introduction to social psychology: a European perspective.

[24] Singer JD, Willett JB (2003). Applied longitudinal data analysis: modeling change and event occurrence.

[25] Steketee G, Chambless DT (1992). Methodological issues in prediction of treatment outcome. Clin Psychol Rev.

[26] Taylor GJ, Bagby RM (2004). New trends in alexithymia research. Psychother Psychosom.

[27] Timbremont B, Breat C (2002). Children’s Depression Inventory. Handleiding Nederlandse versie.

[28] Wright M, Banerjee R, Hoek W, Rieffe C, Novin S (2010). Depression and social anxiety in children: differential links with coping strategies. J Abnorm Child Psychol.

